# Visual Behaviours (ViBes) in Cerebral Visual Impairment: Validating a Descriptive Tool to Support Diagnosis and Monitoring

**DOI:** 10.22599/bioj.290

**Published:** 2023-06-14

**Authors:** Rachel F. Pilling, Louise Allen, Pamela Anketell, Raimonda Bullaj, Janet Harwood, Suzanne Little

**Affiliations:** 1 University of Bradford, UK; 2 University of Liverpool, UK; 3 University of Ulster, UK; 4 University College London, UK; 5 CVI Society, UK

**Keywords:** paediatric ophthalmology, cerebral visual impairment, cortical visual impairment, assessment, diagnosis

## Abstract

**Introduction:**

Cerebral visual impairment (CVI) is the most common cause of visual impairment in children in the UK. Diagnosis is based on identification of visual behaviours (ViBes) relating to visual dysfunction. Examination techniques and inventories have been developed to elicit these in children with a developmental age of two years or more. The absence of a structured approach to recording visual behaviours in children with complex needs is a barrier to diagnosis. The aim of the study was to develop a matrix of visual behaviours seen in pre-verbal and pre-motor children with visual impairment and establish its content validity and inter-rater reliability.

**Methods:**

**ViBe content validation:**

Visual behaviour descriptors relating to visual function were collated and categorised by expert consensus of vision professionals into a matrix composed of three functions (attention, field/fixation, motor response) and five levels (0 = no awareness; 1 = visual awareness; 2 = visual attention; 3 = visual detection; 4 = visual understanding).

**ViBe inter-rater reliability:**

The participants (two orthoptists, an optometrist, an ophthalmologist and two qualified teachers of the visually impaired) used the ViBe matrix to independently score each of 17 short video clips of children demonstrating visual behaviours seen in CVI.

**Results:**

The ViBe matrix will be presented. Cohen’s kappa for the matrix was 0.67, demonstrating moderate-to-strong inter-rater reliability.

**Conclusion:**

The development of standardised descriptors can support clinicians and teachers in identifying areas of concern for children with complex needs. In addition, the ViBe matrix could be utilised in research, clinical and diagnostic reports to clearly communicate the areas of visual dysfunction and track progress resulting from interventions.

**Key Points:**

- The absence of a structured approach to recording visual behaviours in children with complex needs is a barrier to diagnosis.
- The ViBe matrix offers descriptors relating to visual behaviours and has demonstrated acceptable inter-rater reliability.
- The tool may support the identification and diagnosis of cerebral visual impairment in a population of children who cannot access standard testing.

## Introduction

Cerebral visual impairment (CVI) is the most common cause of visual impairment in children in the UK (Teoh, Solebo & Rahi 2021b). Aside from reduced acuity, symptoms typical of cerebral visual impairment include visual field defects, oculomotor disorders, impaired motion perception and visuo-cognitive or visuoperceptual impairments (Williams et al. 2021). Diagnosis is based on identification of verifiable visual dysfunction (Sakki et al. 2018)—that is, a health professional confirming the presence of atypical visual behaviours (ViBes) which occur due to abnormal visual processing (Ortibus, Fazzi & Dale 2019; Lueck, Dutton & Chokron 2019; Williams et al. 2021; McConnell, Saunders & Little 2021). Traditional acuity tests, assessment batteries and validated psychometric testing useful in the diagnosis of CVI have been the focus of several recent studies (Ortibus, Fazzi & Dale 2019; Donaldson et al. 2019; Woodhouse et al. 2014). However, these are often inaccessible for children with complex and multiple disabilities. Various inventories and questionnaires have been developed to elicit these behaviours, but these again require the child to have a level of motor and/or verbal developmental age of two years or more (Macintyre-Beon et al. 2012; Tsirka et al. 2020; Fazzi & Micheletti 2020; Ortibus et al. 2011; Vancleef et al. 2020; Ben Itzhak et al. 2020).

Undiagnosed CVI within the special school population has been reported, resulting in children with visual dysfunction not being offered or unable to access the support they need to maximise learning opportunities (Pilling & Outhwaite 2017). It is known that risk factors for CVI are also associated with developmental delay (Salt & Sargent 2014), for example, cerebral palsy (Pagliano et al. 2007), hypoxic ischaemic encephalopathy, prematurity (Dutton 2013) or genetic/chromosomal disorders (Bosch et al. 2016). It is widely accepted that for this cohort, a pivotal element in the diagnosis of CVI is a period of observation of the child using their vision (Philip et al. 2016). However, the absence of a structured approach to recording visual behaviours in children with complex needs is emerging as a barrier to diagnosis.

The aim of the study was to develop a matrix of visual behaviours (ViBes) seen in children with emerging language and/or gross motor skills with an associated visual impairment and to establish its content validity and inter-rater reliability amongst eye health professionals and teachers of the visually impaired.

## Method

The study was in two parts: content validity and inter-rater reliability.

### ViBe content validation

**Figure 1:**
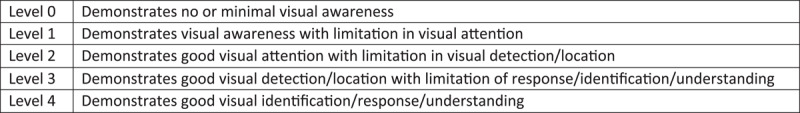
Levels of visual behaviours (ViBe).

A pool of 28 visual behaviour descriptors was collated from a review of relevant studies and personal practice (see Table 1). (Ben Itzhak et al. 2020; Rossi et al. 2017; Lee et al. 2021; Hall Lueck & Dutton 2015; Lueck, Dutton, & Chokron 2019; Pilling & Little 2019; Baranello et al. 2020) The statements were reviewed by six experts in visual assessment of children with CVI, including a paediatric ophthalmologist, two orthoptists, one optometrist and two qualified teachers of the visually impaired, each with several years’ experience of assessing children with complex needs and CVI. Participants were asked to include, amend, combine or delete items from the list. The resultant set of 33 ViBe descriptors (Table 2) were presented to participants in a random order. Participants were independently asked to categorise each statement to indicate the level of visual awareness they would consider each visual behaviour represented (Figure 1, adapted from Hall Lueck and Dutton (2015)). Consensus was defined as agreement by the majority of participants.

**Table 1:** Initial visual behaviour descriptors presented to participants to include, amend, combine or delete.


1	Intentional visual avoidance (deliberately looks away from a presented target)

2	Fleeting and random visual attention

3	Reaction to the same stimulus fades with repeat showing/slower with repeat testing

4	Visual attention suppressed in a specific area of visual field (e.g., right, left, inferior)

5	Shift of visual attention from near to distant

6	Field of visual attention globally suppressed

7	Consistent vision switch on in response to visual stimulus and then sustained for a period of seconds

8	Gazing and scanning and expressing side preference

9	Roving eye movements

10	Appears aware of faces, large objects

11	Vision switched on in response to bright light

12	Smiling or frowning to light movement

13	Fixation difficult to obtain, of short duration (<3 s)

14	Light gazing (at ceiling or window)

15	Repeat short fixations in the direction of the stimulus

16	Using peripheral field of vision rather than central field of vision

17	Visual alertness improves in dimly lit room

18	Looks away from object as motor response occurs

19	Delay in visual/motor response to object/light

20	Looks away from object as motor response occurs

21	Change in head position—side looking to locate object or place it in area of visual field where attention best

22	Eccentric fixation

23	Head movement to follow an object instead of eye movement

24	Swiping at or inaccurate grapsing of an object

25	‘Stilling’ or cessation of movement or vocalisation in response to visual stimulus

26	Vision occasionally present, but is suppressed during other sensory tasks

27	Brief eye contact, not during speech

28	Vision sometimes present, but suppression of other senses during visual tasks


**Table 2:** Visual behaviour descriptors presented to participants to categorise into levels of visual function.


1	Intentional visual avoidance (deliberately looks away from a presented target)

2	Visual awareness* fleeting or not directly linked to a stimulus

3	Visual attention* reduced in a specific area of visual field (e.g., inferior, right) as compared with remainder of field

4	Visual attention* restricted to a small area of visual field (noted by gazing or scanning into that area)

5	Preference shown for visual attention* in one hemifield

6	Visual attention* seemingly equal in all areas of visual field

7	Does not promptly shift visual fixation from one object to a new object presented in another area of accessible visual field

8	Intentionally changes head/eye position to maximise area of best visual attention*

9	Sphere of visual attention* present at less than 1 m; unable to detect new objects beyond 1 m

10	Sphere of visual attention* beyond 1 m and used to locate new objects

11	Promptly shifts visual fixation from one object to a new object presented in another area of the accessible visual field

12	Visual attention* switched on (e.g., fixation, tracking, stilling) in response to an audio or visual stimulus and held for up to 3 seconds

13	Visual attention* (e.g., fixation, tracking, stilling) shown in response to an audio or visual stimulus and remains on to detect new stimuli

14	Visual attention* seemingly on all the time

15	Roving eye movements

16	Shows awareness* of large, non-illuminated objects in close proximity (e.g., face, ball)

17	Shows awareness* of a bright light

18	Delayed fixation on an object, lasting <3 seconds

19	‘Light gazing’ in the direction of ceiling lights or window

20	Preference for use of peripheral vision over central vision; adopts atypical head or eye position in response to visual stimulus

21	Visual awareness* improved in dim light vs bright or room light

22	Upper limb motor response markedly delayed >5 seconds and/or gross

23	Upper limb motor response slightly delayed <5 seconds and/or inaccurate

24	Fixation on object lost during upper limb motor response

25	Immediate upper limb motor response

26	Accurate upper limb motor response

27	Moves head to locate an object rather than using ocular movement/fixation

28	‘Stilling’ of sensory self-stimulation in response to a visual stimulus

29	Improvement in visual response when removed from a stimulating environment (e.g., reduced clutter, reduced noise)

30	Able to name, sign, match or indicate recognition of object

31	Vision appears switched on all the time

32	Vision appears more ‘on’ than ‘off’

33	Vision appears more ‘off’ than ‘on’


* **Awareness** may be shown by a change in body position, head position, stilling, verbalisation, smile or pupil reaction. The position change need not be in the direction of the object. Visual awareness stops short of fixation on the object. * **Visual attention** may be shown by an ocular movement or motor response directed at the object, including fixation, tracking or eye movement to relocate an object. * **Motor response** may be shown by ‘reach and grab’. * **‘Stilling’** refers to the cessation of a movement (e.g., rocking, chewing, flapping) or vocalisation (e.g., humming, lip smacking, speech) as a response to a visual stimulus.

### ViBe inter-rater reliability

To ascertain inter-rater reliability, 17 video clips of children exhibiting CVI-related behaviours were curated from a selection available in the public domain. The clips were between 30 and 150 seconds in length. Videos were selected to ensure all visual behaviours described within the ViBe matrix were present in at least one video clip; in most instances, clips contained several visual behaviours. The curated videos were sourced from CVI specialist websites and peer-reviewed publications describing the assessment of children with CVI. They showed children with a confirmed diagnosis of CVI or children demonstrating normal visual behaviours as part of an infant developmental assessment. The clips were edited and uploaded to a video-viewing platform to eliminate any possibility of participants viewing comments or additional footage which might influence their scoring. The same participants (four eye health professionals and two visual impairment teachers) were invited to view the video clips and use the ViBe matrix to assign an overall ViBe score. Participants viewed the clips independently of each other and were allowed to watch the video clips more than once. Paired responses (based on order of receipt from participants) were analysed using Cohen’s kappa (McAlinden, Khadka & Pesudovs 2011).

## Results

**Figure 2:**
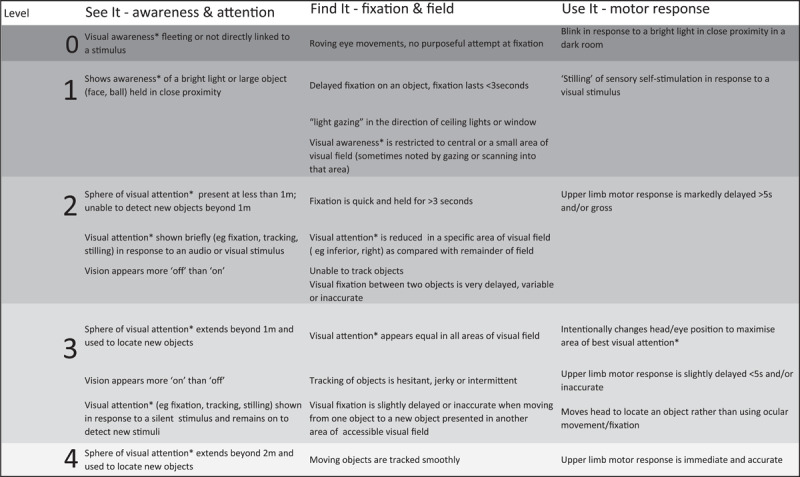
The ViBe Matrix.

Consensus on level of visual awareness was reached for 28 items. Five items did not reach consensus, as participants noted these could appear at any level of visual function and perhaps represented an environmental factor which might contribute to visual performance (e.g., improvement in visual response when removed from a stimulating environment, such as by reducing noise or clutter). Rather than exclude these from the matrix, they have been retained to facilitate appreciation of these common responses and the impact of a child’s surroundings on their function. Items were subsequently presented in three columns to allow a structured approach during testing or observation to elicit various visual behaviours. The three categories (awareness and attention, fixation and field and motor response) represent the general functions of vision. They were chosen to mirror terminology emerging within the visual impairment education community—See It, Find It, Use It—to describe ‘how’ a child uses their vision in everyday practice. The use of this structure has been shown to facilitate understanding and communication of the nature of CVI to a novice audience (paper in press). The terms are non-hierarchical—that is, a child may have normal acuity (see it—awareness and attention) and normal understanding in order to make a response (use it—motor function) but have significant difficulties tracking moving objects or using eye movements efficiently to search a room or page for objects which are not in their central vision (find it—fixation and field). Another child may have difficulty in holding visual attention for more than a few seconds (see it—awareness and attention) but, once switched on, be able to track objects or find a new object presented in a different area of their visual field (find it—fixation and field) and respond appropriately (use it—motor response). The resulting ViBe matrix is shown in Figure 2.

The results of video assessment using ViBe matrix are shown in Table 3. Cohen’s kappa for the matrix was 0.67, demonstrating moderate-to-strong inter-rater reliability.

**Table 3:** Results of video clip ViBe score.


VIDEO CLIP/PARTICIPANT OVERALL VIBE SCORE	SCORER A	SCORER B	SCORER C	SCORER D	SCORER E	SCORER F

Clip 1	1	1	0	0	1	1

Clip 2	1	2	2	2	2	2

Clip 3	3	3	3	3	3	3

Clip 4	0	0	1	1	1	0

Clip 5	2	2	2	2	3	3

Clip 6	1	1	1	2	2	2

Clip 7	2	2	2	3	3	3

Clip 8	3	3	3	3	3	4

Clip 9	3	2	3	2	2	3

Clip 10	2	1	1	2	2	2

Clip 11	2	1	0	1	1	1

Clip 12	2	2	3	3	3	3

Clip 13	3	3	3	3	4	3

Clip 14	4	3	4	4	4	4

Clip 15	2	1	2	2	2	3

Clip 16	1	1	1	1	2	2

Clip 17	1	1	0	1	2	1


## Discussion

A key barrier in the diagnosis of CVI in children with complex needs is the paucity of formal assessment. Parents have highlighted that among the barriers to obtaining diagnosis and support for CVI, the absence of formal documentation of function visual assessment is a key issue (Goodenough, Pease & Williams 2021). A recent publication examining temporal trends in the epidemiology of childhood sight impairment identified that ‘tackling cerebral visual impairment is now the biggest challenge and biggest opportunity for reducing the burden of childhood blindness’ (Teoh, Solebo & Rahi 2021a).

There are three elements to the diagnosis of CVI: a risk factor (e.g., developmental anomaly), an observed or reported visual dysfunction and atypical visual function detected on examination (Pilling et al. 2022; Boonstra et al. 2022). The ViBe matrix is a tool which uses a blend of qualitative descriptors and a quantitative score to enable professionals to record a child’s visual behaviours as part of the diagnostic process.

A strength of the study is the participation of a range of professionals involved in the care of a child with complex needs. The development of a tool which can be used across health and education will facilitate the shared care of children with CVI, enabling clear communication between services.

Our study is limited by the use of short video clips rather than live participants. This method was chosen to allow each participant to see exactly the same visual behaviours and minimise the variability inherent in the assessment of children with complex needs on different days or times. The study design also did not allow for participants to interact with the child in order to draw out visual behaviours, an approach which is intuitive to them.

We acknowledge the limitation of video clips in demonstrating interactive visual behaviours. We attempted to overcome this by ensuring each behaviour was present in at least one video, and it is reassuring that within each domain (attention and awareness; field and fixation; motor response) the full range of scores (0–4) were utilised by each participant. It is possible that bias may have been introduced, with participants scoring sympathetically or harshly. However, all but one participant used the full range of scores (0–4) in their overall assessment. We acknowledge that all participants involved in this study had extensive experience of CVI. Future studies to examine the validity of the ViBe matrix in clinical and ‘live’ educational settings are planned to involve professionals with a broader range of experience in assessing children with complex needs.

It has been reported that in children with early-onset CVI, around a third of children are unable to complete standard testing protocols due to developmental delay. Children in this group were most likely to have CVI clinically confirmed, using observation techniques and parental reporting (Sakki et al. 2020). The ViBe matrix provides a structure and language which can aid reporting and communication of atypical visual behaviours and be utilised as part of a suite of assessments in children with complex needs, moving families one step closer to diagnosis.

## Data accessibility statement

The data that support the findings of this study are available from the corresponding author upon reasonable request.
